# Thick blood film examination for *Plasmodium falciparum *malaria has reduced sensitivity and underestimates parasite density

**DOI:** 10.1186/1475-2875-5-104

**Published:** 2006-11-08

**Authors:** Philip Bejon, Laura Andrews, Angela Hunt-Cooke, Frances Sanderson, Sarah C Gilbert, Adrian VS Hill

**Affiliations:** 1 Centre for Clinical Vaccinology and Tropical Medicine, University of Oxford, Oxford, OX3 7LJ, UK; 2 Wellcome Trust Centre for Human Genetics, University of Oxford, Roosevelt Drive, Oxford OX3 7BN, UK

## Abstract

**Background:**

Thick blood films are routinely used to diagnose *Plasmodium falciparum *malaria. Here, they were used to diagnose volunteers exposed to experimental malaria challenge.

**Methods:**

The frequency with which blood films were positive at given parasite densities measured by PCR were analysed. The poisson distribution was used to calculate the theoretical likelihood of diagnosis. Further *in vitro *studies used serial dilutions to prepare thick films from malaria cultures at known parasitaemia.

**Results:**

Even in expert hands, thick blood films were considerably less sensitive than might have been expected from the parasite numbers measured by quantitative PCR. *In vitro *work showed that thick films prepared from malaria cultures at known parasitaemia consistently underestimated parasite densities.

**Conclusion:**

It appears large numbers of parasites are lost during staining. This limits their sensitivity, and leads to erroneous estimates of parasite density.

## Background

Microscopy of thick blood films is the usual diagnostic test for *Plasmodium falciparum *malaria. Density is usually assessed by thick films, either by counting parasites per microscope field, or by counting parasites per hundred white blood cells [1]. Thick films contain several layers of red cells, whereas thin films contain a single layer of spread red cells. Thus, for a fixed number of microscope fields, thick films allow the microscopist to examine a larger number of red cells for the presence of parasites, and low parasitaemias can be more readily identified by thick film. Thin films are preferred to examine the morphology of parasites and determine species. Non-immune individuals may be unwell when one parasite or less is present in an entire thick film, requiring laborious, repeated examinations to make a diagnosis.

Sporozoite challenge experiments were conducted, where volunteers were exposed to experimental malaria challenge to assess candidate malaria vaccines [2-5]. Treatment decisions were based on blood film analysis, but PCR was conducted on all blood samples [6]. PCR data was not made available until after the trial was been completed.

The sensitivity of thick blood films was studied using data obtained during these trials, compared this with quantitative PCR data, and further investigated these findings with *in vitro *studies.

## Methods

### Sporozoite challenge

Volunteers gave informed consent. Procedures were reviewed by OXREC (Oxford Research Ethics Committee), the local ethics committee, and were in accordance with the declaration of Helsinki (revised 1983). Twice daily blood samples were taken from day 6 until day 14, then daily until day 21. At least 100 high powered fields of a thick blood film were viewed and quantitative PCR performed on each sample. Volunteers were treated when a single parasite was seen by blood film, after the appearance of the parasite was confirmed by a second microscopist. Neither managing clinicians nor microscopists were aware of PCR data during the trial.

### Thick blood films

Giemsa staining was used for the first two sporozoite challenge studies, and Field's stain in coplin jars for the later two studies. The thick film was air dried in both methods. For giemsa staining, the film was stood in 5% Giemsa for 30 minutes, then washed gently in tap water and air dried. Field's stain was applied by dipping the slide into Field's stain A for 3 seconds, then into tap water for 3 seconds (with gentle agitation), into Field's stain B for a further 3 seconds and then washing gently in tap water to remove excess stain. The slide was then air dried for at least 30 minutes. The lead microscopist held a post in the London School for Hygiene and Tropical Medicine Clinical Parasitology Laboratory, the UK national reference laboratory, and others at the Medical Research Council, the Gambia. The lead microscopist examined slides produced by serial dilutions, blind to source. The average thick film uses 10 μl of blood spread over one thousand high powered fields, so the 100 high powered fields routinely examined during views 1 μl of blood [7].

### PCR

The PCR method is described elsewhere [6]. Briefly, EDTA anticoagulated blood samples were filtered to remove leukocytes, DNA was purified from 0.5 ml filtered blood, and eluted into 50 μl. A portion of the multicopy 18S (small subunit) ribosomal RNA genes of *P. falciparum *was amplified by PCR and the increase in PCR product detected by binding the fluorescent dye SYBR Green I using the Rotor-Gene Real-Time PCR machine (Corbett Research), using l μl extracted DNA in duplicate. The increase in PCR product is quantitated by comparison with standard preparations of known parasite numbers.

870 paired blood films and PCR samples were examined from 80 volunteers with rising parasite counts.

## Results

### Sensitivity of blood films during sporozoite challenge

The poisson distribution was used to calculate the likelihood of sampling a parasite within the blood volume examined in microscopy, at given parasite densities identified by PCR. At low parasitaemias there was a discrepancy between the likelihood of diagnosis calculated by PCR readings, and the actual frequency of diagnosis at that density by thick film. For the thick films prepared between 100 and 1000 parasites per ml, the overall calculated likelihood of sampling a parasite in 1 μl was 26%. However, thick films had a sensitivity of only 9% (95% CI 4–15%) in this range. Between 1,000 and 10,000 parasites per ml, the calculated likelihood of a parasite being present was 84%, but the actual sensitivity of thick films was 29% (CI 19–39%). It was only above 10,000 parasites per ml when thick films had higher sensitivity (81% CI 65–97%), when the calculated probability of sampling was 99%.

This surprising finding suggested that a significant number of parasites were not visualized on a thick film despite being theoretically present in the original blood sample used to make the film. Results did not vary according to staining protocol (Giemsa or Field stain) or by microscipist. A similar density threshold for reliable diagnosis of malaria by thick film examination is reported elsewhere [8].

### Serial dilution

Experiments using serial dilution of a known parasite density were then conducted to extend this observation. An *in vitro *culture of *Plasmodium falciparum *was prepared at 5–10% parasitaemia. The parasite count was first accurately determined by a thin film (in duplicate). The culture was then serially diluted with uninfected, fresh whole blood. Red cell counts were made by Coulter™ counter for both the original culture and the uninfected blood used for serial dilutions. This allowed accurate calculation of predicted parasite numbers, without having to count thin films at each dilution. At each serial dilution, PCR analysis was conducted on 0.5 mls blood, and 2 thick films made, using exactly 10 μl. The whole film was read, blind to source.

### Densities seen by blood film during serial dilution

**Figure 1:**
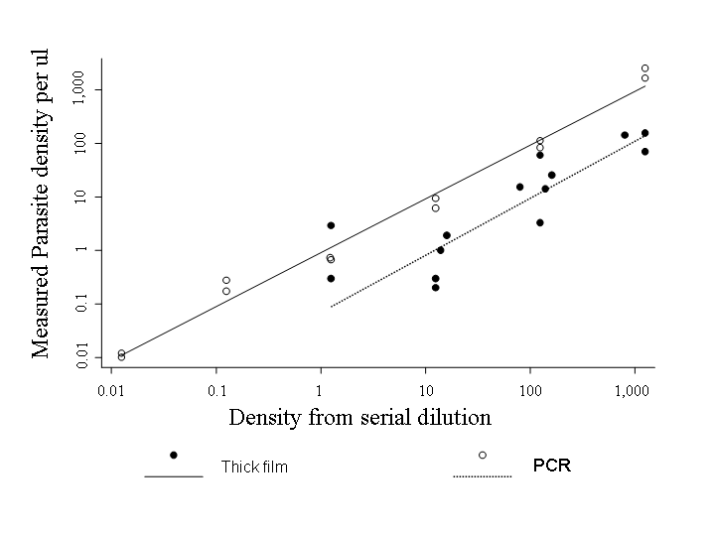
At each serial dilution (x axis), parasite densities seen by PCR (open circles) and parasite densities seen by thick blood film examination (filled circles) are both plotted on the y axis. The PCR readings are the result of a single experiment, the thick film readings are the results of two experiments. The solid line (least squares regression line for PCR results against densities known from serial dilution) is given by y = 0.98x + 0.07 (95% CI -0.12 to 0.27). The dotted line (regression line for thick film densities against serial dilution, ignoring the outlier) is given by y = 0.78x - 1.5 (95% CI -0.61 to -2.4) (y = 0.81x - 1.26, 95% CI - 0.07 to -2.5 including the outlier). Densities measured by thick film are therefore approximately 1 log lower than those calculated by serial dilution, whereas PCR readings match the serial dilution more closely.

For the serially diluted parasite cultures, the parasite density measured by PCR and thick film was compared with that calculated from serial dilution (Fig 1). Although PCR readings corresponded well with actual parasite numbers generated from serial dilutions, thick films were less reproducible, but tended to measure parasite densities approximately one log lower than those calculated by serial dilution.

## Discussion

It is unlikely that cells or parasites are hidden during microscopy, since adequate preparation of the slide ensures visibility through all planes of focus. It is more likely that parasites are either washed off or lysed during staining, since PCR of the staining reagents to detect parasites was positive, and transfer of parasites from positive slides to negative slides during staining has been recognized for some time [8].

Thin film and thick film parasite density estimates have been compared in previous studies. Although thick films are more sensitive than thin films, they significantly underestimated the parasite density in some studies [7,9,10], but not in others [11]. These previous studies lacked accuracy, since at high parasitaemias thick films are difficult to count accurately, and thin films cannot be counted accurately at low parasitaemias. In the study presented here, this difficulty was avoided by using serial dilution to provide known concentrations of parasites, and the accuracy of serial dilution was confirmed by quantitative real time PCR. PCR counts gene copy number, and this might have led to an over-estimate of parasite numbers when counting multi-nucleated schizonts. However, only mononucleated parasites are identified in peripheral blood at the low parasitaemias seen in this study, and *in vitro *cultures were synchronous.

## Conclusion

Thick films are considerably less sensitive than might be possible and underestimate parasite densities. In routine clinical work in non-endemic areas the loss in sensitivity makes thick film examination much more laborious, and a fixation method that prevented parasites being washed off would improve the sensitivity of the method. In malaria endemic areas, the loss in sensitivity is not critical, since semi-immune patients are likely to have a high parasitaemia if they present with febrile malaria. In epidemiological studies, parasite density thresholds are defined to distinguish febrile malaria from chronic parasitaemia [12,13]. A systematic underestimate of densities would not alter the classification of individuals, but if some density counts are made by thick and some by thin film, and this will lead to an underestimate of the lower parasitaemias counted by thick film. Furthermore, should epidemiological studies to define parasite densities be repeated by quantitative PCR, considerably higher parasite density thresholds will be identified to define malaria cases. These will not be comparable to previous studies.

## Authors' contributions

Experimental Design: PB, FS, SG

Experimental work: LA, FS, AHC.

Writing of Manuscript PB, SG, AVSH.
